# Magnetic molecules as local sensors of topological hysteresis of superconductors

**DOI:** 10.1038/s41467-022-31320-5

**Published:** 2022-07-04

**Authors:** Giulia Serrano, Lorenzo Poggini, Giuseppe Cucinotta, Andrea Luigi Sorrentino, Niccolò Giaconi, Brunetto Cortigiani, Danilo Longo, Edwige Otero, Philippe Sainctavit, Andrea Caneschi, Matteo Mannini, Roberta Sessoli

**Affiliations:** 1 Department of Industrial Engineering (DIEF) and INSTM Research Unit, University of Florence, Via Santa Marta 3, 50139 Florence, Italy; 2 Institute for Chemistry of Organo-Metallic Compounds (ICCOM-CNR), Via Madonna del Piano 10, 50019 Sesto Fiorentino (FI), Italy; 3 Department of Chemistry “U. Schiff” (DICUS) and INSTM Research Unit, University of Florence, Via della Lastruccia 3-13, 50019 Sesto Fiorentino (FI), Italy; 4 Synchrotron SOLEIL, L’Orme des Merisiers, 91192 Saint-Aubin, France; 5 Institut de Minéralogie, de Physique des Matériaux et de Cosmochimie (IMPMC), CNRS, Sorbonne Université, 4 place Jussieu, 75252, Paris, Cedex 5 France

**Keywords:** Electronic devices, Superconductors, Magnetic properties and materials, Surface spectroscopy

## Abstract

Superconductors and magnetic materials, including molecules, are key ingredients for quantum computing and spintronics. However, only a little is known about how these materials interact in multilayer nanostructures like the hybrid architectures nowadays under development for such advanced applications. Here, we show that a single layer of magnetic molecules, Terbium(III) bis-phthalocyaninato (TbPc_2_) complexes, deposited under controlled UHV conditions on a superconducting Pb(111) surface is sensitive to the topology of the intermediate state of the superconductor, namely to the presence and evolution of superconducting and normal domains due to screening and penetration of an external magnetic field. The topological hysteresis of the superconducting substrate imprints a local evolution of the magnetisation of the TbPc_2_ molecules in the monolayer. Element and surface selective detection is achieved by recording the X-ray magnetic circular dichroism of the Tb atoms. This study reveals the impressive potential of magnetic molecules for sensing local magnetic field variations in molecular/superconductor hybrid devices, including spin resonators or spin injecting and spin filtering components for spintronics applications.

## Abstract (web summary)

Magnetic molecules have long been seen to hold promise in magnetic sensing applications. In this paper, Serrano et al show that a single layer of a magnetic molecule, a terbium based complex, is sensitive to the local magnetic field variation of a superconducting surface on which it is deposited.

## Introduction

Recently, the coupling between magnetic materials and superconductors (SCs) has raised an increasing interest for its potential in spintronics and quantum technologies^1,2^. First, it enhanced spintronic-related properties, such as spin injection and magnetoresistance^1^. At the nanoscale, the interaction of single spins with superconducting substrates revealed the appearance of local bound states within the SC band gap^3,4^, opening a route to creating Majorana bound states of relevance as fundamental units for topological quantum computing applications^5^. Differently from individual atoms or bulk impurities, magnetic molecules profit from a well-defined chemical structure that can be engineered to tune the coupling strength between molecular spin and superconducting substrates and thus control the local bound states^4^. From a technological point of view, the realisation of hybrid molecular—superconductor architectures can permit the integration of molecular qubits in quantum circuits, such as superconducting microwave resonators^6,7^, or to produce dissipationless spin currents through spin singlet-to-triplet conversion mechanism^8^.

An important step forward in the research on hybrid materials comprising molecules and superconductors was achieved by depositing one layer of single molecule magnets (SMMs) retaining their magnetic memory on Pb(111). On one hand, it was shown that the transition of Pb to the superconducting state alters the magnetisation dynamics of the Fe_4_ SMMs complexes inducing a switching of their magnetic state from a blocked state to a resonant regime via quantum tunnelling of the magnetisation^9^. This phenomenon was observed for the first time in the magnetic hysteresis loop of the SMMs layer recorded by synchrotron radiation and indicated that the superconducting transition severely influences the magnetic properties of the molecular film at a large scale. On the other hand, this observation suggested that magnetic molecules can be used as local sensors of the superconductive state. Indirect sensing of the superconducting state is commonly achieved by depositing an inorganic ferromagnetic layer and following its magnetic evolution by X-ray absorption^10^ or magneto-optical (MO) methods^11–13^. Other than the intrinsic spatial resolution limit of the specific probe, local sensing is fundamentally limited by the response being mediated by the correlation of the ferromagnetic layer. On the contrary, nanometre-sized magnetic molecules in direct contact with the surface give an independent response, pushing the sensing limit to the nanoscale.

**Fig. 1:**
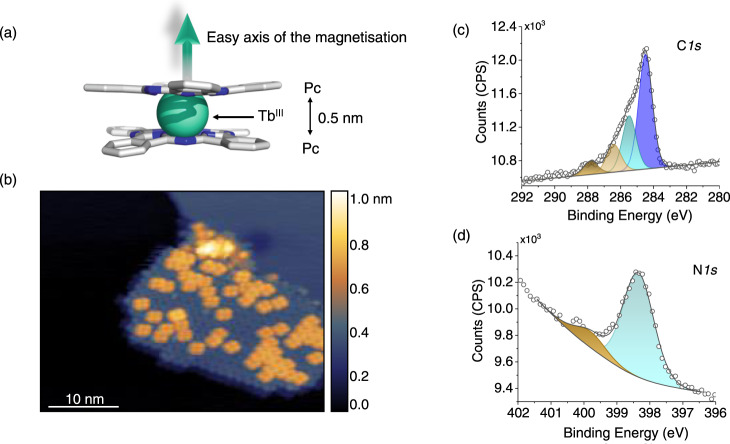
Structural characterisation of the TbPc_2_ sub-monolayer on Pb(111). **a** Scheme of the TbPc_2_ structure. Colour code: grey, carbon; blue, nitrogen; light cyan, terbium; hydrogens are omitted for clarity. The easy axis of the magnetisation is directed perpendicularly to the phthalocyanine (Pc) planes and sketched by an arrow. **b** STM image of a TbPc_2_ island on Pb(111) recorded at 35 K (I_tunnel_= 5 pA, V_bias_= 2 V). The Z-colour scale is shown on the right. **c** C*1s* and **d** N*1s* XPS core-level spectra of the TbPc_2_ sub-monolayer on Pb(111). Main components are shown in blue or cyan; shake-up components are shown in brown.

To further explore the potential of magnetic molecules as sensors, here we have grown on top of a Pb(111) single crystal a layer of an highly anisotropic SMM, the Terbium(III) bis-phthalocyaninato (TbPc_2_) complex, that is known to significantly interact with the metallic surfaces^14–16^. TbPc_2_ is a double-decker system formed by two phthalocyanines (Pc) coordinating a Tb^III^ ion^17–21^ (Fig. 1a). The complex is characterised by strong uniaxial anisotropy with the easy axis of the magnetisation oriented perpendicularly to the Pc planes (Fig. 1a). Near liquid helium temperatures and in bulk crystals, the large energy barrier separating the ground doublet states (J_z_ = ±6) hinders the reversal of the magnetisation, and TbPc_2_ behaves as a single molecule magnet. At ariance with Fe_4_ SMM complexes^22,23^, both the electronic and magnetic properties of TbPc_2_ are particularly sensitive to the interaction with substrates. The signature of this interaction was observed for a wide range of metallic and non-metallic substrates either at the single molecule level or at the larger scale of the molecular film^24–26^. Remarkable effects were found in the magnetic hysteresis loop of TbPc_2_ films, which is quenched for molecules directly interacting with metals while preserved or even enhanced by using decoupling layers^25–27^ or anchoring groups^28^. Small openings of the hysteresis loop were found for TbPc_2_ monolayer on TiO_2_ films^29^, graphene^25^, or HOPG^30^ substrates. On the other side, MgO films separating the TbPc_2_ from the metal Ag(100) surface, led to large magnetic remanence and disappearance of the quantum tunnelling of the magnetisation at zero field^26^.

This sensitivity of TbPc_2_ films to the environment has been exploited here to investigate the magnetisation of TbPc_2_ at the interface with Pb(111) and across its superconducting transition, as a function of the temperature and applied magnetic field. To achieve this purpose, we deposited a sub-monolayer of TbPc_2_ molecules by thermal sublimation on the Pb(111) surface. The study, performed by *in house* surface characterisation methods (*e.g*. Scanning Tunnelling Microscopy, STM, and X-ray Photoelectron Spectroscopy, XPS) and synchrotron radiation, showed that the magnetisation curve of the investigated TbPc_2_ sub-monolayer is influenced by the topology of the superconducting and normal domains of Pb when the magnetic field intensity is raised or lowered. This effect causes the opening of the hysteresis loop of TbPc_2_, reflecting the topological hysteretic behaviour of the superconducting substrate.

## Results and discussion

The Pb(111) crystal was prepared according to the procedure reported in the Methods and preliminarily characterised by XPS and STM (see Fig. S1). The bare Pb(111) surface shows large terraces with monoatomic steps of 0.3 nm height^9^ (see Fig. S1e). The molecular deposit was obtained dosing a sub-monolayer amount of thermally sublimated TbPc_2_ molecules (see Methods). The growth and adsorption configuration of TbPc_2_ on Pb(111) was studied by STM at low temperature (35 K) and low surface coverage (~ 30% of a complete monolayer). The STM image of Fig. 1b shows an island of TbPc_2_ molecules assembled on the Pb(111) surface with some second layer molecules adsorbed on top. The formation of regular islands is common for TbPc_2_ thin films on many other surfaces^26,31,32^. The height of the TbPc_2_ island is about 0.33 nm ± 0.02 (see line profile in Fig. S2). This value is strongly related to the magnitude of surface-molecule interaction and was found to be 0.25 nm on Ag(111)^33^ and 0.4 nm on Au(111)^31,34^. This indicates that weak molecule-substrate interaction is present. STM images also show that molecules maintain the lying-down configuration (Pc planes parallel to the surface), usually observed on metal surfaces^25,26,31–34^.

A TbPc_2_ sub-monolayer (~ 80%) on Pb(111) was characterised by XPS at room temperature to study the molecular stoichiometry and the interaction with the substrate (see Methods), evidencing spectral features (Fig. 1c, d and Fig. S2) in line with those observed for monolayer and thick films on other surfaces^35–37^. The C*1s* spectrum of the TbPc_2_ film on Pb is characterised by two major components at 284.5 eV and 285.4 eV ascribed to C-C and C-N bonds, and related shake-up signals at 286.4 eV and 288.0 eV^35,36^. The N*1s* spectrum shows the main component at 398.5 eV and a shake-up at 400.4 eV^35–37^. Both C*1s* and N*1s* have similar shapes and energies compared with signals from the massive phase and with the same molecule deposited on other surfaces^28,29,35,36^, indicating that the weak surface-molecule interaction does not cause significant charge transfer effects. This is at variance with what it was observed on a TiO_2_ rutile single crystal where a strong surface-molecule interaction led to a marked energy shift of the N*1s* and C*1s* components ascribed to molecular oxidation^35^. The C/N XPS signal ratio is 3.8, in close agreement with that expected from the molecular stoichiometry (C/N_theory_ = 4), and indicates the integrity of the molecular layer. In Figure S2, the XPS Pb*4f* spectrum after molecular deposition shows the Pb*4f*_*7/2*_ signal calibrated at 136.9 eV, and the Tb*3d*_3/2_ signal at 1276 eV^9,36,38,39^. We remark that the Tb*3d*_5/2_ signal overlaps with the Auger lines of Pb and C, α-N_6_O_4,5_O_4,5_ and KVV^40^, respectively, and it cannot be used for quantitative analysis.

**Fig. 2:**
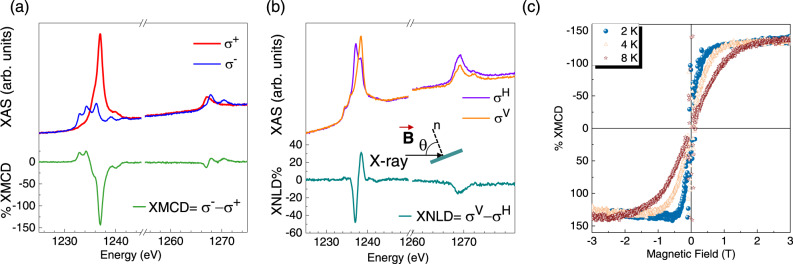
Magnetic characterisation of the TbPc_2_ monolayer on Pb(111). **a** XAS and XMCD spectra recorded at θ = 0°, 2 K and 3 T. **b** XAS and XNLD spectra recorded at θ = 45°, 2 K and 3 T. The inset depicts the detection geometry having the X-ray beam axis and applied magnetic field oriented at an angle θ from the sample’s surface normal, n. **c** Magnetic hysteresis loop recorded at θ = 0° and at different temperatures.

Synchrotron characterisation was performed to investigate the magnetic properties of the TbPc_2_ sub-monolayer (~ 80%) on Pb(111) by X-ray Absorption Spectroscopy (XAS), see Fig. 2a–c. All data were acquired in the Total Electron Yield (TEY) mode (see Methods) with the X-ray beam axis and applied magnetic field at an angle θ from the sample’s surface normal (see inset Fig. 2b). XAS spectra were recorded using the positive (σ^+^) and negative (σ ^–^) circular polarisation of the X-ray light to obtain the X-ray Magnetic Circular Dichroism (XMCD) spectrum (σ ^–^ - σ ^+^) at the Tb M_4,5_ edges. Fig. 2a shows the XAS and XMCD spectra recorded at normal incidence (θ = 0°), 2 K and 3 T. The XMCD intensity value is normalised to the edge jump value of the isotropic XAS spectrum (see Methods). The strong dichroic signal at the M_5_ edge, 1237 eV, also persisting at 4 K and 8 K (see Fig. S3b, c), has an intensity of about 140%, in good agreement with the previously investigated TbPc_2_ monolayers and thick films^24–28^. Indeed, this XMCD spectrum is representative of saturated Tb^III^ ions with total angular momentum J = L + S = 6 and in the symmetry imposed by the two Pc ligands^24,41^. At θ = 45°, the intensity of the XMCD signal slightly decreases to about 120% (see Fig. S3a) as a consequence of the uniaxial anisotropy of TbPc_2_ molecules that are oriented with their easy axis of magnetisation perpendicular to the surface^24^.

At θ = 45°, XAS spectra were also recorded using linear vertical (σ ^V^) and horizontal (σ ^H^) light polarisation, whose difference (σ^V^ – σ^H^) provides the X-ray Natural Linear Dichroism (XNLD) spectrum. The XNLD contribution gives information about the orientation of TbPc_2_ molecules on the surface^24,25^. The XNLD spectrum for the TbPc_2_ sub-monolayer on Pb(111) is shown in Fig. 2b. The shape of the dichroic signal, expressed in percentage according to the formula reported in the Methods, presents a minimum at 1237 eV with an intensity of about −50%. This confirms the lying-down orientation of the molecules in the monolayer with a narrow distribution of the orientation, similarly to highly oriented TbPc_2_ films on graphene and gold substrates^24,25^.

Magnetic field dependence of the XMCD intensity at the Tb M_5_ edge, 1237 eV, and at the pre-edge, 1225 eV, were measured to detect the evolution of the normalised XMCD while sweeping the magnetic field (see Methods) and follow the magnetic behaviour of the absorber. Figure 2c shows the trend obtained by applying the magnetic field perpendicularly to the sample surface (θ = 0°) with a scan rate of 2 T/min from −3T to 3 T and vice versa. The figure shows the temperature dependence of these loops at 8 K, 4 K, and 2 K (the error on the temperature value is estimated to be less than 10%). As expected, given the magnetic anisotropy of the investigated molecular layer, decreasing the temperature increases the steepness of the curves at low fields and decreases the saturation field. At 2 K, the dichroism at saturation is about 140%, coherently with the intensity of the maximum dichroic signal in the XMCD spectrum recorded at 3 T and shown in Fig. 2a. The expected opening due to the SMM behaviour is quenched at all temperatures, and TbPc_2_ molecules behave as paramagnets. This behaviour is typical of monolayers of TbPc_2_ deposited on metals^16,24,26,27,42^. However, the interest in this magnetic molecule/superconductor hybrid resides in the sensitivity given by the strong uniaxial magnetic anisotropy of the TbPc_2_ complex to the superconducting transition.

The Pb(111) substrate is superconductive below its critical temperature, *T*_*C*_ = 7.2 *K*, and within the critical field value *H*_*C*_ that varies with the temperature according to the formula ${H}_{C}(T)={H}_{C}(0)[1-{(\frac{T}{{T}_{C}})}^{2}]$^43^. Being  *H*_*C*_(0) = 0.08 *T* for Pb^44^, one expects here *H*_*C*_ = 0.074 T. Thus, in the previous measurements (Fig. 2c) the magnetic field region within *H*_*C*_ is hidden below the limit of the scan resolution adopted and below the strong noise of the XMCD signal detected by the TEY when the field is swept across zero.

**Fig. 3:**
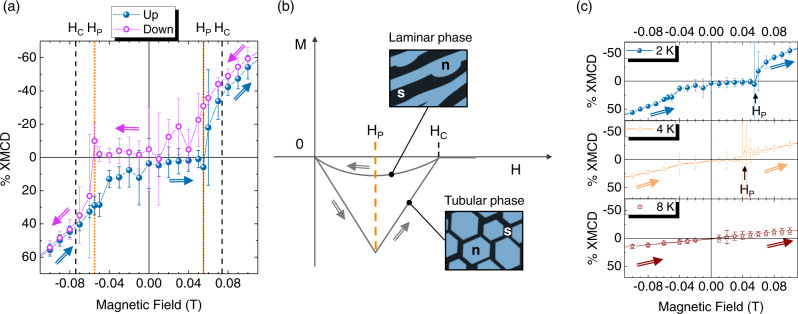
Magnetic behaviour of the TbPc_2_ sub-monolayer on Pb(111) across the superconducting transition. **a** Magnetisation curves of TbPc_2_ monolayer on Pb(111) at 2 K and θ = 0° within the critical field of the superconductor (*H*_*C*_). The complete magnetic field screening effect of Pb is observed for increasing magnetic field intensity within a certain field H_P_. The arrows indicate the field scan direction. **b** Sketch of a typical magnetisation loop of disk shaped Pb single crystals (see ref. ^12^). The hysteresis loop of bulk Pb crystals originates from the different topology of the intermediate state when the magnetic flux penetrates or is expelled from the substrate. The topology (tubular and laminar) of the micrometric superconducting (***s***) and normal (***n***) domains in the intermediate state depicted in the figure is representative of the magnetooptical images of Pb crystals of ref. ^12^ **c** Magnetisation curve (up branch) of TbPc_2_ on Pb(111) for θ = 0° at different temperatures below (2 K and 4 K) and above (8 K) the critical temperature. Error bars in panels a and c indicate the Standard Deviation (SD) of the signal at each field (see Methods).

To overcome this limitation, magnetisation curves were collected for each X-ray light polarisation by time-integrating the TEY signal at each sampled field (see Methods) instead of acquiring the TEY signal during a magnetic field sweep. Figure 3a shows that at 2 K, the trend of the TbPc_2_ XMCD signal within the superconducting regime of Pb, is strongly influenced and presents a hysteretic behaviour. We observe that by decreasing the magnetic field intensity below $|{H}_{C}^{Pb}(2)|=0.072\,{{{{{\rm{T}}}}}}$, the XMCD signal of TbPc_2_ follows an almost linear trend. In this region, the effect of the superconducting transition in the TbPc_2_ magnetisation curve is therefore negligible, indicating that the magnetic field is only partially screened by the superconductor. However, after crossing zero field and increasing the magnetic field intensity, the XMCD values are zero (within the error limits) until a specific field |*H*_*P*_| = 0.055 T. This suggests that the magnetic field is completely screened out by the Pb substrate below |*H*_*P*_|. By increasing the field above *H*_*P*_, the XMCD values suddenly jumps to a finite value as the superconductor is back in a regime where the external magnetic field is only partially screened before it turns into the normal state at |*H*_*C*_|.

The fact that the TbPc_2_ XMCD signal of Fig. 3a shows a hysteretic behaviour is the signature of the transition of the Pb(111) crystal to the superconducting state and—in particular—of the different topology of the normal (***n***) and superconducting (***s***) domains when the magnetic flux is penetrating or exiting the superconductor.

It is well-established that type I superconductors, below their critical field, *H*_*C*_, and critical temperature, *T*_*C*_, exhibit an intermediate state (IS) in which both ***s*** (where the magnetic field is completely expelled) and ***n*** domains (where the magnetic field penetrates) coexist at the micrometric scale^12,13,43,45–47^. We remark that the X-ray beam used for the measurements of Fig. 3a hits at the centre of the crystal with a spot diameter of about 800 μm thus averaging over *s* and *n* domains. The topology, *i.e*. the shape of these domains, strongly differs during the increase (magnetic flux penetration phase) or decrease (magnetic flux expulsion phase) of the magnetic field intensity. This effect was observed in MO images for various type I SCs metals^45,48^, including Pb crystals^12,13,43^. The combination of MO and SQUID measurements demonstrated that the topological variation of the IS domains causes a hysteretic behaviour (topological hysteresis)^12,13,43^. In particular, Prozorov^12^ treated the case of disk-shaped Pb crystals (of shape and dimensions comparable with the Pb substrate used here) in an axial magnetic field, *i.e*., along the cylinder axis.

A clear interpretation of the TbPc_2_ hysteresis at 2 K shown in Fig. 3a can be indeed achieved by considering the magnetisation curves of TbPc_2_ film in light of the magnetisation curves of Pb crystals reported by Prozorov^12^ and here sketched in Fig. 3b. By decreasing the field below *H*_*C*_, *i.e*. during the magnetic flux expulsion, the magnetisation curve of the Pb crystal shows small magnetisation absolute values, poorly affecting the magnetisation of TbPc_2_ whose trend is only slightly deviated from linearity below *H*_*C*_ (see Fig. 3a). According to Prozorov^12^, in these conditions, the **s** and **n** domains of the Pb crystal show a laminar topology (see the sketch in Fig. 3b). The magnetic flux can exit the sample only through the ***n*** domains, which shrink on lowering the magnetic field intensity until reaching zero field, where the substrate is completely superconducting, and the magnetic flux is completely excluded (Meissner state). It is worth noticing that when the intermediate state has a laminar topology, the magnetic flux is never fully screened by the superconductor until the external magnetic field is zero. By increasing the external magnetic field intensity from zero, the magnetic flux does not penetrate the bulk of the sample, which remains in an almost diamagnetic state until *H*_*P*_ (negative linear slope in Fig. 3b)^12,49^. In these conditions, magnetic flux can only enter at the edges of the Pb disks but cannot migrate towards its centre^12,49^, which justifies the overall zero XMCD values detected on the TbPc_2_ layer when the field is increased from zero to |*H*_*P*_| = 0.055 T. When magnetic field is increased above *H*_*P*_, magnetic flux penetrates through ***n*** domains that expand from the sample edges over the whole substrate with a tubular topology, where a hexagonal symmetry is favoured (see the sketch in Fig. 3b)^12^. In this phase, TbPc_2_ molecules acting as sensors provide an increasing XMCD signal due to the expansion of the ***n*** domains (Fig. 3a). We remark that in the intermediate state the magnetic field intensity in normal regions is always equal to *H*_*C*_^43,49^ and that the increase of the XMCD intensity here detected is only due to the average over ***n*** domains of increasing extension.

Furthermore, we notice that the XMCD error bar amplitude is maximum at the onset of field penetration in tubular topology; this effect is not observed at the entrance of the SC state (field exclusion onset), where TbPc_2_ is already magnetised. The same behaviour was found in the hysteresis curve of Fe_4_ complexes on Pb^9^. We ascribe it to the abrupt change in the local magnetic induction of the TbPc_2_ molecules felt by photoelectrons and secondary electrons of the TEY signal when exiting the demagnetised condition^9^.

The hysteretic behaviour of the TbPc_2_ XMCD signal shown in Fig. 3a is maximum at *H*_*P*_. The XMCD values are null at zero field, compatibly with the topological hysteresis curves of the SC and further indicating that the hysteresis loop is not a consequence of flux pinning inside the superconductor^12^. We remark that the topological hysteretic behaviour of Pb could not have been observed with Fe_4_ complexes, due to their SMM properties^9^. The strong drop of the Fe_4_ magnetisation value observed during magnetic field screening*, i.e*. lowering the magnetic field intensity below *H*_*C*_, was a consequence of the increasing fraction of magnetic molecules sitting on ***s*** regions and undergoing fast demagnetisation due to resonant quantum tunnelling of the magnetisation in zero local field. Furthermore, Fe_4_ molecules could not be sensitive to the onset of field penetration (and exit from the superconducting state) since the system remained frozen in the non-magnetic state until the first field-induced level crossing was reached, thus reopening the tunnelling channel^9^. On the contrary, the paramagnetic character of TbPc_2_ allows an enhanced sensitivity to the magnetic flux variation on the surface of the superconductor.

A closer look at the magnetisation curve of Fig. 3a further supports our interpretation. Indeed, the onset of the field penetration inside the superconductor varies with the geometry of the superconducting sample, which determines its demagnetising factor (N). Being *H*_*P*_ = (1–*N*)*H*_*C*_^12,43^ and considering the experimental *H*_*P*_ value observed in the hysteresis loop at 2 K, we derive an N value for our Pb crystal of about 0.25. This value differs from N ~ 0.5 estimated simply considering the sample geometry (for cylinders, the height-to-diameter ratio);^50,51^ similar discrepancies between the experimental and calculated N value are also observed for the case reported by Prozorov^12^. Even if the derivation of an exact N value is beyond the scope of the present work, it is worth noticing that the different N value observed in our case and in that of Prozorov (being *N* = 0.55)^12^ agrees with the trend expected by the height-to-diameter ratio of the samples.

Figure 3c compares the magnetisation curve of TbPc_2_ on Pb(111)—up branch—recorded at 2 K, 4 K and 8 K. The curves show the magnetic field expelled from the superconductor at negative fields and the magnetic field entry at positive fields. It is evident that the *H*_*P*_ field, *i.e*. the onset of the field penetration, changes with the temperature (at 4 K and 2 K) while it cannot be identified at 8 K since it is above the *T*_*C*_ value. According to the previously reported equations for *H*_*C*_ and *H*_*P*_, and assuming N = 0.25, at 4.2 K, *H*_*C*_ is expected at 0.053 T, and *H*_*P*_ at 0.04 T. The XMCD data of Fig. 3c at 4 K perfectly support this hypothesis, further demonstrating the sensitivity of a single layer of TbPc_2_ molecules to the superconducting transition.

In conclusion, we deposited a single layer of TbPc_2_ molecules by thermal sublimation in ultrahigh vacuum on a clean Pb(111) surface and we characterised the interface properties by XPS and STM to check the molecular film growth and stoichiometry. Synchrotron light was used to address the magnetic behaviour and the molecular ordering of the TbPc_2_ film on a millimeter range by XMCD and XNLD, respectively. X-ray detected magnetisation measurements performed at the Tb edge showed that the TbPc_2_ monolayer behaves as a paramagnet when the substrate is in the normal state, as typically observed for monolayer deposits on metal surfaces. However, an opening of the hysteresis loop was observed below the transition of Pb to the superconducting state. We ascribe this phenomenon to the topological hysteresis loop of the superconducting Pb(111) surface, due to the different shapes of superconducting and normal domains during magnetic field penetration or expulsion. Further, from the analysis of the superconductor hysteresis loop probed by the molecules and its temperature dependence, we derived the critical field and the geometrical factors of the Pb crystal that characterised the transition to the condensate state.

At variance with Fe_4_ SMMs complexes^9^, we showed here that a single layer of magnetic molecules can be sensitive not only to the transition to the superconducting state but also to the topological features of superconducting domains. We stress that such sensitivity was achieved on a single layer of TbPc_2_ in its paramagnetic regime in direct contact with the Pb substrate. TbPc_2_ is a peculiar probe thanks to its strong out-of-plane magnetic anisotropy and its capability to form highly oriented films that make these molecules extremely sensitive to local magnetic flux variation. Our results open the perspective of using the plethora of molecules with spin properties (including highly coherent systems^52,53^) for detecting and investigating superconductivity. We stress that the achievement of sensing magnetic flux penetration in superconductors by magnetic molecules is a great advancement with respect to standard investigation methods by magneto-optics and SQUID magnetometry. First, our method is not a bulk measurement, at variance with SQUID measurements, but a local one. Thus, it is better suited to investigate ultra-thin films that are relevant, for instance, in the investigation of topological superconductivity^54^ and moiré superconductors^55^. Second—though the X-ray detection methods used here give a response that is averaged over hundreds of μm—each molecule behaves as an independent probe of nanometric size, going beyond the resolution limit of both the optical probe and the magnetic correlation in ferromagnetic indicator disks of MO technique. This is highly relevant for the investigation of nanoscale superconductivity, including vortex state motion and confinement^56^.

From another point of view, we also demonstrated that the intrinsic topology of the intermediate state of the superconductor could induce a hysteretic behaviour in an ensemble of paramagnetic molecules. This last outcome might have a great potential for technological applications. Furthermore, these results are significant also for those fields that exploit hybrid molecular/superconductor systems in macroscopic devices, such as resonators^6,7^, or for the detection of localised states occurring at the interface between single spins and superconducting surfaces^4^, where magnetic field screening effects can play an important role.

## Methods

The Pb(111) single crystal was acquired from the Surface Preparation Laboratory (SPL) and it had a disk shape with a diameter of 4 mm and a height of 2 mm. The surface was prepared in ultrahigh vacuum (UHV) by sputtering cycles of Ar^+^ at 1500 eV and annealing at 473 K for 30 min. Before molecular deposition, surface cleanliness was checked by XPS and STM measurements at room temperature (RT). The molecular deposition was performed by thermal sublimation of the molecular powders using a homemade effusion cell. The sublimation temperature of TbPc_2_ was 710 K, and the flux was estimated by a quartz microbalance. Surface coverage was checked by STM using an Omicron VT-STM with a W tip. STM measurements were carried out by cooling down the sample to 35 K with a helium flux to reduce molecular mobility on the surface.

XPS measurements were carried out using a monochromatic Al Kα radiation (hν = 1486.6 eV, SPECS mod. XR-MS focus 600) and a SPECS Phoibos 150 1DLD electron analyser mounted at 54.44° with respect to the X-ray beam. XPS measurements were performed at normal emission with the pass energy set to 40 eV. Spectra were analysed using the CasaXPS software and calibrated at the Pb*4f*_*7/2*_ signal at 136.9 eV (see Fig. S2, ESI). Spectra were fitted with a linear or tougard background^57^, and single-peak components were deconvoluted using a mixed Gaussian and Lorentzian function (70/30). All the above-mentioned *in house* characterisations were performed on the very same sample without breaking the vacuum.

X-ray absorption spectroscopy (XAS) experiments were carried out at the DEIMOS beamline, synchrotron SOLEIL (Saint-Aubin, France)^58^. All samples were prepared and characterised in Florence and transferred to the end-station using a UHV suitcase (P_base_ = 5 ∙ 10^−10^ mbar). XAS spectra were recorded in total electron yield (TEY) mode^59^ using both linear and circularly polarised light^59^ in a temperature range between 8.0 ± 0.2 and 2.0 ± 0.1 K and magnetic field within ± 3 T (measurements’ parameters are specified in the text). XAS spectra were recorded at the Tb M_4,5_ edges at angles θ, defined as the angle between the **k** X-ray propagation vector and the surface normal. XMCD spectra were normalised to the M_5_ edge jump of (*σ* ^+^ + *σ* ^−^)/2 and expressed in percentage (% XMCD), while XNLD spectra at θ=45° were normalised to the M_5_ edge jump of the isotropic spectrum (*σ*^iso^ =1/3*σ* ^V^ + 2/3*σ* ^H^) and expressed in percentage (% XNLD)^60^. XAS and dichroic spectra were calibrated by setting the maximum of the XMCD and XNLD at the Tb M_5_ edge to 1237 eV. Magnetic hysteresis curves were obtained by recording the field dependence of the % XMCD at the Tb M_5_ edge (measured at 1237 eV and here referred to the pre-edge dichroism at 1225 eV) sweeping the magnetic field between −3 T and 3 T with a scan rate of 2 T/min. Data treatment for XMCD, XNLD, and hysteresis was performed employing pyDichroX^61^. To avoid the noise present in the TEY signal during the field sweeping when the magnetic field is close to zero, the magnetic hysteresis at low field intensities (Fig. 3) were acquired by recording the TEY signal at each sampled field, and for each X-ray light polarisation (circular left and right) using a time-scan acquisition (scan time 120 s with 0.2 s of integration time for a total of 600 data points). This procedure was adopted for both edge and pre-edge energies to get the normalised dichroic signal. The XMCD values and the associated error bars (calculated as the standard deviation, SD) reported in the hysteresis of Fig. 3 were then obtained considering the average TEY signal at each field, polarisation, and energy, and the relative SD.

## Supplementary information

Supplementary Information

## Data Availability

The STM, XPS and synchrotron datasets generated and processed during the current study are available in the Zenodo repository, 10.5281/zenodo.6460971.

## Code availability

The code to analyse the XMCD, XNLD and magnetisation data is available in the Zenodo repository, 10.5281/zenodo.6458173.
