# Supplementary material for: Magnetic molecules as local sensors of topological hysteresis of superconductors
Source: Nat Commun. 2022 Jul 4;13:3838. doi: 10.1038/s41467-022-31320-5 (PMC9253336; doi:10.1038/s41467-022-31320-5)
Supplement: Supplementary file 1 — Supplementary Information [file 41467_2022_31320_MOESM1_ESM.pdf]

Supplementary Information

Magnetic molecules as local sensors of topological hysteresis of superconductors

Giulia Serrano,[1*] Lorenzo Poggini,[2] Giuseppe Cucinotta,[3] Andrea Luigi Sorrentino[1,3], Niccolò Giaconi,[1,3] Brunetto Cortigiani,[3] Danilo Longo,[4] Edwige Otero,[4] Philippe Sainctavit,[4,5] Andrea Caneschi,[1] Matteo Mannini,[3*] Roberta Sessoli[3]

[1] Department of Industrial Engineering (DIEF) and INSTM Research Unit, University of Florence, Via Santa Marta 3, 50139 Florence, Italy. giulia.serrano@unifi.it

[2] Institute for Chemistry of Organo-Metallic Compounds (ICCOM-CNR), Via Madonna del Piano 10, 50019 Sesto Fiorentino (FI), Italy.

[3] Department of Chemistry "U. Schiff" (DICUS) and INSTM Research Unit, University of Florence, Via della Lastruccia 3-13, 50019 Sesto Fiorentino (FI), Italy. matteo.mannini@unifi.it

[4] Synchrotron SOLEIL, L'Orme des Merisiers, 91192 Saint-Aubin, France.

[5] Institut de Minéralogie, de Physique des Matériaux et de Cosmochimie (IMPMC), CNRS, Sorbonne Université, 4 place Jussieu, 75252 Paris Cedex 5, France.

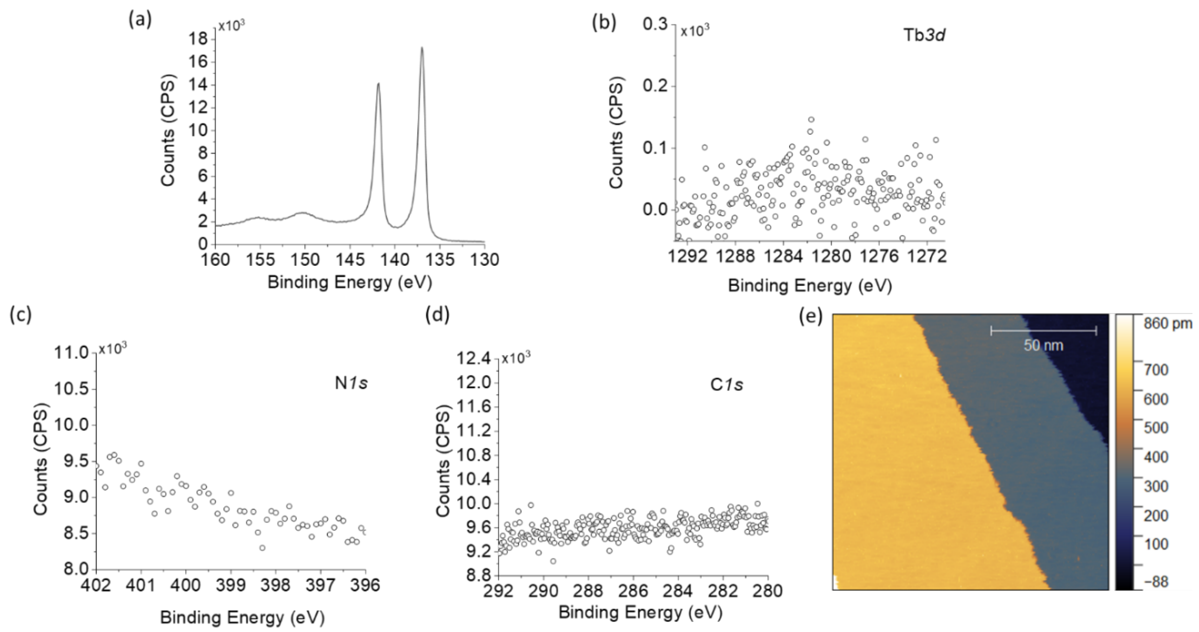

Figure S1. Pb*4f* (a), Tb*3d* (b), N*1s* (c) and C*1s* (d) core-level XPS spectra recorded on the Pb(111) single crystal before the $TbPc_2$ sub-monolayer deposition. (e) STM image of Pb steps recorded at 35K (V_{bias} = 2.0 V, I_{tunnel} = 100 pA).

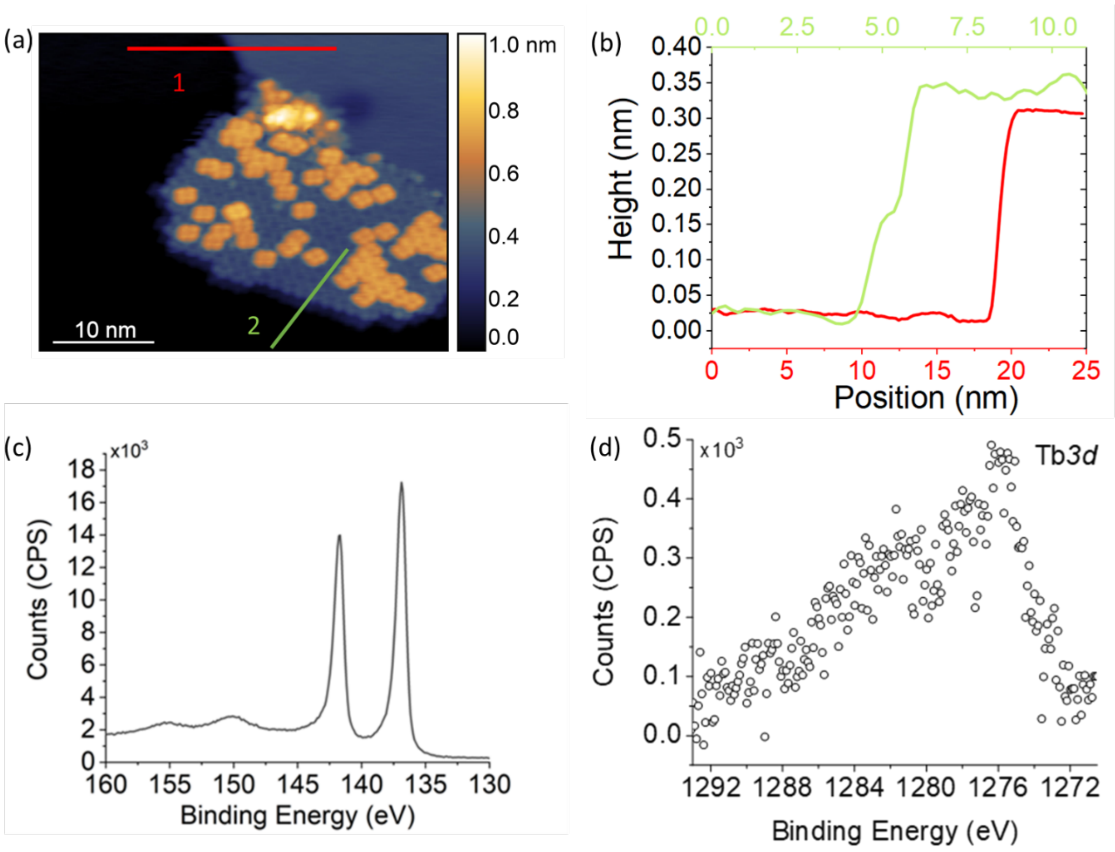

Figure S2. (a)STM image (I = 5pA, V = 2V) at high magnification with profile (b); Pb*4f* (c) and Tb$3d_{3/2}$ (d) core-level XPS spectra recorded after $TbPc_2$ sub-monolayer deposition on Pb(111).

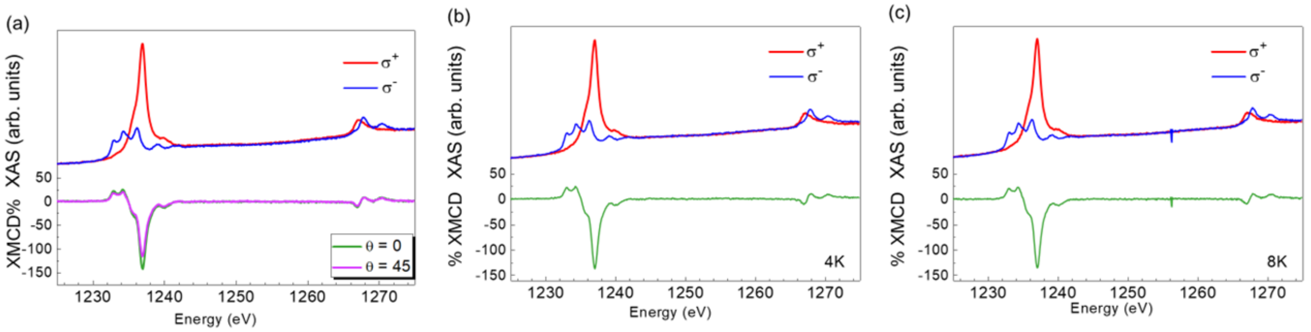

Figure S3. (a) Comparison of the XMCD spectra of the $TbPc_2$ sub-monolayer on Pb(111) at θ = 0º and θ = 45º (see main text), (b) XMCD spectrum at 4 K and (c) at 8 K. All spectra were recorded at 3T.
